# Mfsd2a‐Targeted Therapy for Ischemic Stroke: Mechanisms, Evidence, and Future Prospects

**DOI:** 10.1002/cns.70684

**Published:** 2025-12-08

**Authors:** Zhidong He, Jing Sun

**Affiliations:** ^1^ Department of Neurosurgery China‐Japan Union Hospital of Jilin University Changchun Jilin China; ^2^ Department of Neurology China‐Japan Union Hospital of Jilin University Changchun Jilin China

**Keywords:** blood–brain–barrier, ischemic stroke, Mfsd2a, neuroinflammation, neuroprotection

## Abstract

**Background:**

Ischemic stroke remains a major cause of global disability and mortality, with blood–brain barrier (BBB) dysfunction being a pivotal event in its pathology. Major facilitator superfamily domain‐containing 2a (Mfsd2a), a key lipid transporter at the BBB, has emerged as a promising yet underexplored therapeutic target.

**Objective:**

This review proposes a unifying framework that positions Mfsd2a as a central indicator of ischemic stroke pathophysiology and a potential target for treatment. Although direct clinical evidence remains in its early stages, this review synthesizes foundational knowledge from diverse fields.

**Methods:**

We revisit the established biological functions of Mfsd2a, including its role in inhibiting caveolae‐mediated transcytosis and transporting omega‐3 fatty acids, and detail its core mechanisms in maintaining BBB integrity. This review also correlates these functions with their significant downregulation following ischemic stroke. We then critically evaluate the limited but compelling preclinical evidence from models in which Mfsd2a has been directly targeted and explore innovative therapeutic strategies. Finally, we explicitly address the current limitations, including the scarcity of direct intervention studies, and outline a translational roadmap for future research.

**Results:**

By integrating this dispersed evidence chain, this review aims to solidify the theoretical foundation for Mfsd2a‐targeted therapies and accelerate their clinical development.

**Conclusion:**

Targeting Mfsd2a shows a promising therapeutic strategy to protect the BBB and improve neurological outcomes after ischemic stroke.

## Graphical abstract

This graphical abstract illustrates the central role of Mfsd2a in maintaining blood–brain barrier (BBB) integrity under physiological conditions and its dysregulation following ischemic stroke. In the normal brain (left), Mfsd2a is highly expressed on brain microvascular endothelial cells (BMECs), where it inhibits caveolae‐mediated transcytosis and facilitates the transport of lysophosphatidylcholine‐docosahexaenoic acid (LPC‐DHA), thereby supporting BBB integrity and suppressing inflammation. Following ischemic stroke (right), Mfsd2a expression is downregulated, leading to increased caveolae formation, disruption of tight junctions, BBB breakdown, and enhanced neuroinflammation. The figure also highlights the promising therapeutic strategies aimed at restoring Mfsd2a function, including gene therapy, nutritional intervention with LPC‐DHA, upstream regulatory approaches, and pharmacological activation.

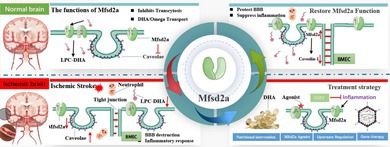

## 1 Introduction

Ischemic stroke is caused by the interruption of blood supply to the brain, resulting in cerebral ischemia, anoxia, and necrosis, and also triggering a cascade of pathological events. Although thrombolysis and endovascular mechanical thrombectomy are effective means of vascular recanalization, there are strict time window–limitations and a risk of hemorrhagic transformation caused by the destruction of the blood–brain barrier (BBB) after reperfusion. Therefore, a significant proportion of patients may be unable to benefit or could face serious complications [1, 2, 3, 4]. Therefore, developing therapeutic strategies that extend beyond vascular recanalization to protect the neurovascular unit (NVU), particularly by stabilizing the BBB, represents an urgent unmet need in stroke research [5, 6, 7].

The BBB composed of highly specialized brain microvascular endothelial cells (BMECs), strictly controls the entry and exit of substances both in and out of the brain parenchyma through supporting structures such as tight junctions (TJs), adhesion junctions, pericytes, and astrocyte endopods. After a stroke, ischemia/reperfusion injury rapidly destroys TJ proteins, activates endothelial cell inflammatory pathways, and induces the upregulation of matrix metalloproteinase (MMP) expression, ultimately leading to BBB leakage [6, 8, 9]. BBB leakage not only aggravates vasogenic cerebral edema and promotes the infiltration of inflammatory cells but also enables entry of harmful substances into the brain parenchyma and also significantly increases the risk of hemorrhagic transformation after thrombolysis [2, 10]. Therefore, protecting and repairing the BBB is key requirement for reducing secondary injury after stroke, expanding the treatment window, and improving the prognosis of patients [11, 12]. Because brain endothelial cells are the most important components of the BBB, their protein expression is a key target for the treating BBB [13, 14]. Mfsd2a, a key transmembrane lipid transporter, facilitates the transport of DHA in the form of lysophospholipids and participates in the dynamic regulation of BMECs [15, 16]. According to recent studies, Mfsd2a is an upstream regulator of Cav‐1 and has been identified as an important component of the formation and integrity of BBB [17]. In this context, Mfsd2a as a key molecule in BBB endothelial cells, provides a novel approach for stroke treatment through its functional regulation. This review aims to elucidate the role of Mfsd2a in ischemic stroke pathophysiology and evaluate the therapeutic potential of strategies targeting its function. We also propose a unifying framework in which Mfsd2a serves as a central node in ischemic stroke pathology. Its downregulation acts as a master switch, simultaneously disabling the transcytosis inhibitor, compromising tight junction stability, and depriving the brain of anti‐inflammatory DHA, thereby initiating a synergistic cascade of BBB disruption and secondary neuronal injury.

## 2 Structure and Physiological Functions of Mfsd2a

The unifying framework proposed earlier in the introduction section—positioning Mfsd2a as a central node in stroke pathology—rests upon its fundamental biological roles. Therefore, a precise understanding of the physiological functions of Mfsd2a is essential for understanding how its dysfunction drives ischemic injury. Mfsd2a is uniquely and highly expressed in the endothelial cells of BBB, blood–retinal barrier, and blood–labyrinth barrier, with minimal expression in the peripheral vasculature [16, 18, 19]. Structural studies have revealed its characteristic bowl‐like extracellular domain, which specifically recognizes the phosphatidylcholine head of LPC, and a sodium‐dependent transport mechanism that drives the conformational changes necessary for substrate uptake [20, 21]. Its two interconnected core functions—the maintenance of BBB integrity via the suppression of transcytosis and the regulation of brain lipid homeostasis—are severely disrupted following cerebral ischemia. Elucidating these functions provides the essential framework for understanding its potential as a potent therapeutic target.

**FIGURE 1:**
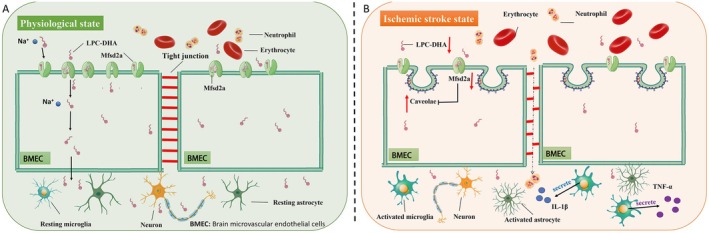
Role of Mfsd2a in maintaining blood–brain barrier (BBB) integrity under physiological conditions and its dysfunction after ischemic stroke. (A) Physiological state: Mfsd2a is highly expressed in healthy brain microvascular endothelial cells (BMECs). Driven by sodium ions, Mfsd2a facilitates the uptake of lysophosphatidylcholine (LPC)‐conjugated lipids (e.g., LPC‐DHA) into the brain parenchyma. This suppresses the caveolin‐1 (Cav‐1)‐dependent transcytosis pathway. Together with intact tight junction proteins, this ensures a low‐permeability BBB, which restricts the entry of immune cells (e.g., neutrophils) and maintains internal environmental homeostasis, thereby supporting normal neuronal function. (B) Ischemic stroke state: In response to ischemic insult, Mfsd2a expression and function are significantly downregulated. The loss of Mfsd2a‐mediated suppression leads to a marked increase in Cav‐1‐dependent transcytosis. Concurrently, tight junctions are disrupted, and proinflammatory cytokines (e.g., TNF‐α) are released. These changes result in a leaky BBB, allowing for the extravasation of neutrophils and other blood components into the brain and exacerbating neuronal damage and inflammation. BMEC, brain microvascular endothelial cell; Cav‐1, caveolin‐1; LPC, lysophosphatidylcholine; LPC‐DHA, lysophosphatidylcholine‐docosahexaenoic acid; TNF‐α, tumor necrosis factor‐alpha.

In central nervous system (CNS), the role of Mfsd2a extends far beyond mere solute transport; Mfsd2a acts as a central hub for maintaining NVU homeostasis and BBB integrity (Figure 1A). As a key inhibitor of caveolae‐mediated transcytosis, the functional expression of Mfsd2a can significantly inhibit caveolin‐1‐mediated transcytosis in endothelial cells, thereby reducing BBB permeability [17, 22, 23]. Perhaps the most therapeutically relevant function of Mfsd2a in stroke is its role as a master inhibitor of caveolae‐mediated transcytosis. Mfsd2a transport activity, rather than the protein itself, creates a lipid environment within the endothelial cell membrane that is unfavorable for the formation and fission of caveolae vesicles [15]. The incorporation of transported LPC‐DHA into the membrane increases its fluidity and disrupts the stability of specific membrane microdomains known as lipid rafts, which are essential platforms for the assembly of caveolae [15, 24]. This transport activity underlies the suppression of nonselective transcytosis. This mechanism is of direct pathophysiological significance because the downregulation of Mfsd2a following ischemic insult (as detailed in the following section) directly lifts this inhibition, leading to a well‐documented surge in caveolae‐mediated transcytosis. This surge represents the initial phase of BBB opening, allowing uncontrolled passage of plasma proteins and potentially harmful substances into the brain parenchyma [23, 25]. Recent work has further solidified this link, demonstrating that protective interventions, such as the administration of glial growth factor 2 (GGF2), exert their beneficial effects on the BBB in stroke models, specifically by upregulating Mfsd2a and thereby suppressing the Cav‐1‐mediated transcytosis pathway [23]. Furthermore, the role of Mfsd2a in barrier integrity extends to the paracellular pathway. The DHA‐containing phospholipids transported by Mfsd2a are integrated into the phospholipid bilayer, increasing its fluidity. This optimized membrane environment is crucial for the correct localization, conformational stability, and dynamic assembly of tight junction complexes. This mechanistic insight is strongly supported by experimental evidence showing that inhibition of Mfsd2a leads to a significant reduction in the expression and proper localization of key tight junction proteins, such as ZO‐1 and occludin [19]. Critically, the disruption of Mfsd2a function, as occurs during stroke, compromises this vital support system for the paracellular seal.

The second core function of Mfsd2a is its role as the primary transporter for the omega‐3 fatty acid DHA in the form of LPC‐DHA, which is involved in maintaining DHA homeostasis in the brain [26, 27]. This extends beyond a nutritive role to critically determining the brain's resilience to injury. Specifically, DHA is the predominant phospholipid in gray matter membranes and is involved in signal transduction, neurotransmitter release, synaptic plasticity, and neurogenesis. DHA and its derivatives (such as neuroprotectant D1 and resolvin D1) are potent endogenous anti‐inflammatory and proresolving mediators that can actively regulate neuroinflammatory responses [28, 29, 30, 31]. DHA in the brain entirely relies on Mfsd2a to enter the bloodstream as LPC‐DHA. Consequently, Mfsd2a function directly governs brain DHA levels, thereby influencing a broad spectrum of neurological functions and the inflammatory milieu. However, it does not directly function as a classic inflammatory signaling molecule (such as cytokines or chemokines) but rather regulates neuroinflammation through its core lipid transport function, in an indirect but crucial manner. First, Mfsd2a maintains the compactness of the BBB by inhibiting Cav‐1‐mediated transcytosis, making it difficult for immune cells and inflammatory factors in the peripheral circulation to enter CNS [17, 22, 23, 32]. Therefore, when Mfsd2a functions properly, it can physically prevent the invasion of peripheral inflammation and constitute a first line of defense for controlling neuroinflammation. Second, Mfsd2a is the main transporter of omega‐3 fatty acids, especially DHA, is taken up by the brain [15, 16, 27]. After it enters the brain parenchyma, DHA can be metabolized by neurons and glial cells to generate a series of specific proresolving mediators (SPMs), which are potent endogenous anti‐inflammatory and proinflammatory regression molecules [33, 34, 35]. They can inhibit the excessive activation of microglia, reduce the production of proinflammatory cytokines, and promote the resolution of the inflammatory environment. Therefore, by upregulating the function of Mfsd2a or increasing the supply of LPC‐DHA substrate, the level of DHA in the brain can increase, thereby enhancing the anti‐inflammatory ability of the brain. In addition, many inflammatory signaling receptors [such as Toll‐like receptors (TLRs)] and signal transduction proteins are located in lipid rafts [24]. An increase in the DHA content in the cell membrane can disrupt the stability of lipid rafts, thereby inhibiting the activation of proinflammatory signaling pathways, such as the NF‐κB pathway [24, 28, 33]. From the perspective of membrane biology, Mfsd2a indirectly reduces the inflammatory response of cells by altering the membrane microenvironment. Therefore, the level of functional Mfsd2a directly affects the ability of the brain to resolve and repair antiinflammatory processes, which are essential for recovery from ischemic injury. The downstream consequences of impaired Mfsd2a‐mediated DHA transport are twofold and highly relevant to stroke pathology. A decrease in brain DHA levels leads to insufficient production of SPMs, crippling the endogenous anti‐inflammatory, and proresolving mechanisms of the brain. This creates a permissive environment for sustained microglial activation and production of proinflammatory cytokines, amplifying secondary neuronal damage in the ischemic penumbra. DHA deficiency directly impairs neuronal survival, synaptic function, and plasticity. In the context of ischemia, where neurons are already under metabolic stress, the loss of this crucial lipid support further tips the balance toward cell death.

In summary, Mfsd2a is located at the nexus of BBB integrity and brain lipid homeostasis. Its sodium‐dependent LPC transport activity simultaneously establishes the low‐permeability nature of the BBB and supplies the brain with DHA, a molecule vital for neuroprotection and inflammatory control. The indispensability of these integrated physiological functions makes Mfsd2a a compelling target in ischemic stroke. The significant downregulation of Mfsd2a after ischemia is not a passive epiphenomenon but an active pathological event that unleashes both a breach in the BBB and a failure of endogenous protective systems. The following section will detail the evidence for this dysregulation and its catastrophic consequences.

## 3 Role of Mfsd2a in the Pathophysiology of Ischemic Stroke

Based on the core role of Mfsd2a in maintaining BBB homeostasis mentioned above, Mfsd2a dysfunction has become a key event in the pathology of ischemic stroke (Figure 1B). The temporal pattern of BBB breakdown in stroke is biphasic, with an early phase (hours) dominated by a surge in caveolae‐mediated transcytosis, followed by a delayed phase (days) involving both persistent transcytosis and the disintegration of TJs [36, 37, 38, 39]. Mfsd2a plays a critical role in this specific pathological cascade [25].

The primary and most direct consequence of Mfsd2a downregulation following cerebral ischemia is the disinhibition of caveolae‐mediated transcytosis. The specific mechanism through which Mfsd2a inhibits caveolae is fundamentally tied to its lipid transport function. By continuously transporting LPC‐DHA into the endothelial cell membrane, Mfsd2a enriches the membrane with polyunsaturated fatty acids. This alters the biophysical properties of the membrane, increasing its fluidity and disrupting the stability of cholesterol‐ and sphingolipid‐rich microdomains known as lipid rafts [15, 24]. Because caveolae are a type of lipid raft that requires a specific ordered membrane environment to assemble and pinch off, this Mfsd2a‐mediated lipid remodeling creates an inhospitable membrane landscape, thereby suppressing vesicle formation. In stroke, the significant downregulation of Mfsd2a protein and mRNA expression reverses this suppression. The loss of this inhibitory signal leads to rapid upregulation of caveolin‐1 expression and a dramatic increase in the density of caveolae, unleashing a torrent of nonselective transcellular transport that constitutes the first major breach of the BBB [25]. While the ability of Mfsd2a to suppress transcytosis is a universal principle of BBB maintenance, its paramount importance in ischemic stroke is highlighted by the acute and explosive nature of caveolae‐mediated transcytosis activation in this context. In contrast, in other conditions, such as chronic neurodegenerative diseases or metastatic brain tumors, BBB leakage may develop more insidiously, and the contribution of Mfsd2a dysfunction might be more strongly intertwined with chronic neuroinflammation or sustained DHA deficiency. However, in the acute ischemic cascade, the rapid downregulation of Mfsd2a expression acts as a critical “master switch” that enables immediate, high‐volume transcellular flow, distinguishing it from pathologies where paracellular leakage or chronic barrier deterioration might be the initial dominant feature.

The pathophysiology of Mfsd2a dysfunction in stroke extends beyond the increase in transcytosis inhibition. Two additional interconnected pathways significantly contribute to the damage. The first is the intensification of paracellular leakage. The appropriate membrane fluidity maintained by Mfsd2a‐derived lipids is crucial for the stability and dynamic assembly of tight junction complexes. The downregulation of Mfsd2a compromises this membrane environment, leading to the internalization and degradation of proteins such as ZO‐1 and occludin, thereby directly weakening the paracellular barrier and contributing to the delayed phase of BBB hyperpermeability. Second, the neuroprotective and anti‐inflammatory capabilities are impaired. As the primary transporter of DHA, Mfsd2a dysfunction directly leads to a sharp decrease in brain DHA levels. This deficiency has a dual detrimental effect: it impairs neuronal membrane integrity and synaptic function, reducing resilience to ischemic stress, and it cripples the production of SPMs, which are potent endogenous molecules that halt neuroinflammation and promote repair. Thus, Mfsd2a downregulation not only provides floodgates for peripheral inflammatory cells but also simultaneously disables the key mechanism through which inflammation is resolved in the brain.

In summary, the role of Mfsd2a in ischemic stroke is multifaceted but centered on its core function as a guardian of the BBB. Its significant downregulation acts as a linchpin event, initiating a catastrophic cascade by permitting uncontrolled transcytosis, destabilizing TJs, and crippling endogenous neuroprotection and inflammation resolution. This integrated perspective solidifies its status as a master regulator of stroke pathology and a compelling therapeutic target.

## 4 Preclinical Evidence and Therapeutic Strategies for Targeting Mfsd2a in Ischemic Stroke

Given the core role of Mfsd2a in stroke pathology, targeting this molecule essentially aims to repair the multifunctional protective system proposed in our framework, which has collapsed because of its downregulation. Therefore, the fundamental goal of treatment is to restore or enhance the function of Mfsd2a to simultaneously rescue the integrity of the BBB and reconstruct the anti‐inflammatory environment of the brain. Although direct preclinical evidence from ischemic stroke models remains limited, the existing chain of data—from a solid mechanistic foundation to proof‐of‐concept studies in ischemic models and to highly consistent protective effects in other BBB injury models—strongly supports the feasibility of this treatment strategy.

The most compelling direct evidence comes from a recent study by Zhang et al., which demonstrated that the administration of glial growth factor 2 (GGF2) alleviated cerebral ischemia–reperfusion injury by specifically upregulating Mfsd2a. This intervention effectively suppressed both Mfsd2a/Cav‐1‐mediated transcellular permeability and Pdlim5/YAP/TAZ‐mediated paracellular permeability, leading to significant preservation of BBB integrity and improved outcomes [23]. This study provides a critical theoretical basis for pharmacological targeting of the Mfsd2a pathway as a beneficial strategy in ischemic stroke. Beyond this, evidence from gene therapy approaches also offers further support. Although not exclusively involved in ischemic stroke, Qu et al., showed that adeno‐associated virus (AAV)‐mediated overexpression of Mfsd2a alleviated BBB injury and reversed cognitive dysfunction in a rat model of chronic cerebral hypoperfusion, a key contributor to vascular cognitive impairment and stroke risk [40]. These findings underscore the functional capacity of enhanced Mfsd2a expression to confer neurovascular protection in a related pathological context. To elucidate this limited yet promising direct evidence, we investigated highly consistent findings from other acute CNS injury models in which Mfsd2a has been directly targeted. For instance, in an intracerebral hemorrhage (ICH) model, forced expression of Mfsd2a is sufficient to attenuate BBB disruption by specifically inhibiting vesicular transcytosis [41]. This core finding—that Mfsd2a activation suppresses transcytosis—was directly replicated in a subarachnoid hemorrhage (SAH) model, where AAV‐mediated Mfsd2a overexpression protected the BBB via the same mechanism [42]. Further reinforcing these results, Eser Ocak et al., demonstrated that targeted Mfsd2a overexpression attenuated BBB dysfunction via the Cav‐1/Keap‐1/Nrf‐2/HO‐1 pathway [17]. The consistent success of Mfsd2a‐targeted interventions in stabilizing the BBB across ICH, SAH, and other models, despite differing initial insults, provides robust indirect support for its therapeutic applicability in ischemic stroke. These findings validate that bolstering the Mfsd2a pathway is a generalizable strategy for counteracting BBB failure, a pathological hallmark shared by all these conditions.

**FIGURE 2:**
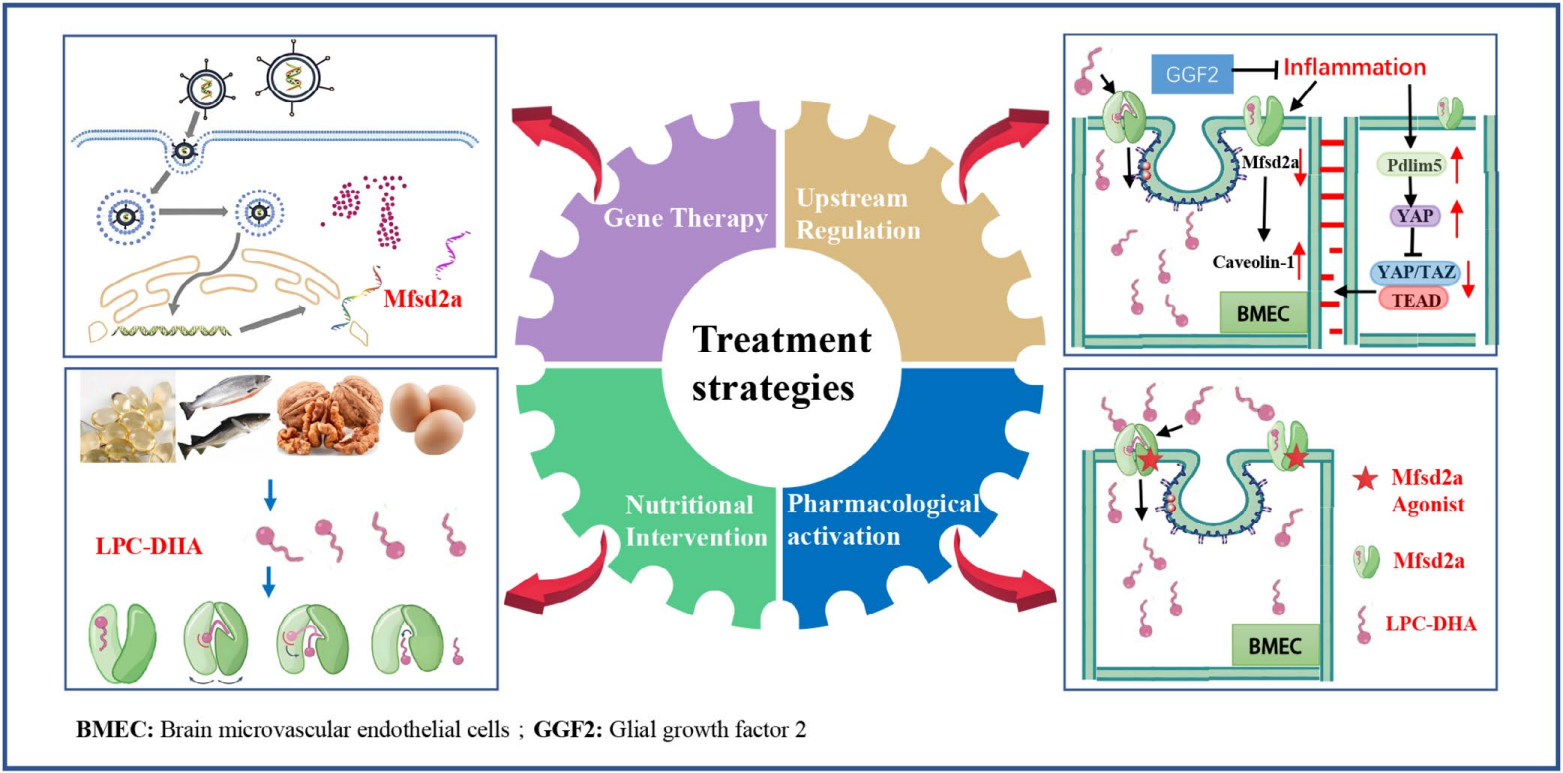
Therapeutic strategies for targeting Mfsd2a in ischemic stroke. Ischemic stroke inhibits Mfsd2a expression and function, leading to blood–brain barrier (BBB) disruption via increased transcytosis and impaired tight junctions. Targeting Mfsd2a presents promising therapeutic strategies: (1) genetic therapy: Viral vector‐mediated (e.g., AAV) overexpression of the Mfsd2a gene; (2) upstream regulation: Glial growth factor 2 (GGF2) and other compounds can upregulate Mfsd2a expression; (3) nutritional intervention: Dietary supplementation with LPC‐DHA; and (4) pharmacological activation: Development of small‐molecule agonists to increase Mfsd2a transport activity. These interventions restore Mfsd2a function, thereby reinforcing BBB integrity, reducing brain injury, and improving neurological outcomes. AAV, adeno‐associated virus; BBB, blood–brain barrier; GGF2, glial growth factor 2; LPC‐DHA, lysophosphatidylcholine‐docosahexaenoic acid.

Building on this mechanistic and evidence‐based foundation, several therapeutic strategies are emerging (Figure 2). As evidenced above, viral vector‐mediated (e.g., AAV) overexpression of Mfsd2a represents a powerful tool for proof‐of‐concept studies, although its clinical translatability for acute stroke remains challenging. The success of GGF2 highlights the potential of identifying molecules that can increase the transcription or expression of Mfsd2a [23]. The search for other small molecules or biologics with this capability is a key research direction. Because the transport of LPC‐DHA by Mfsd2a is the basis for its function, increasing the supply of the substrate LPC‐DHA can theoretically increase its transport efficiency and play a role in the prevention and treatment of stroke [43]. Studies have shown that dietary intake of LPC‐DHA may upregulate the expression of Mfsd2a, serving as a new therapeutic strategy for treating BBB dysfunction in patients with hypoxic–ischemic brain damage [44]. The development of potent, selective, and bioavailable small‐molecule agonists that directly bind to and activate the transport function of Mfsd2a remains the ultimate goal for pharmacotherapy, although no such compounds have yet entered clinical development. In addition, combining Mfsd2a‐targeted therapies with other neuroprotective agents, such as antioxidants, anti‐inflammatory drugs, or antiapoptotic drugs, may also achieve synergistic protective effects, representing a promising area worthy of further investigation. Diverse therapeutic strategies targeting Mfsd2a exhibit varying levels of translational readiness and can be broadly stratified based on current technological and regulatory landscapes. Nutritional supplementation with LPC‐DHA represents the most immediate tractable approach. Given that DHA is an established dietary component, clinical trials evaluating high‐dose LPC‐DHA for acute neuroprotection or chronic supplementation in high‐risk populations could be initiated in the near future. Upstream regulator‐based therapies, exemplified by GGF2, face a more complex path. The preclinical efficacy of GGF2 itself has been demonstrated; however, the development of any novel biologic or small molecule that modulates Mfsd2a expression requires comprehensive investigational new drug (IND)‐enabling studies, followed by phased clinical trials, indicating that the potential clinical application of GGF2 is on a longer horizon. The most direct approach—developing small‐molecule agonists of Mfsd2a—is also the most challenging. The current absence of identified lead compounds, coupled with the inherent difficulties of achieving sufficient brain exposure and ensuring target specificity, means that this strategy constitutes a long‐term goal for the field. Finally, gene therapy using AAV vectors, although representing a powerful proof‐of‐concept tool, is currently less suited for the acute setting of ischemic stroke due to the slow onset of transgene expression and immune‐related challenges. Its application might be reconsidered for preventing recurrent strokes or treating related chronic cerebrovascular diseases.

In conclusion, although the preclinical portfolio of direct Mfsd2a intervention in ischemic stroke is still in its early stages, the existing evidence is compelling. It connects a solid mechanistic basis, a critical proof‐of‐concept study in ischemia, and highly consistent positive results from direct interventions in other BBB injury models. This collective body of work firmly positions Mfsd2a as a promising and legitimate target for future therapeutic exploration in ischemic stroke. The primary challenges going forward will be the development of specific pharmacological agents and the validation of these strategies in comorbid animal models that more closely reflect the clinical reality of stroke patients.

## 5 Challenges and Future Perspectives

Despite the compelling mechanistic rationale for targeting Mfsd2a, this field is not without its challenges, which present critical avenues for future investigation. Most notably, as correctly highlighted by the need for more research, the number of preclinical studies that directly modulate Mfsd2a specifically in ischemic stroke models remains limited. Although the foundational evidence from other BBB injury models is strong, dedicated validation in well‐characterized, comorbid stroke models (e.g., aged, hypertensive, or diabetic animals) is urgently needed to enhance translational relevance. A paramount hurdle is the development of potent, selective, and bioavailable Mfsd2a‐targeting pharmacologic agents, which is still in its infancy. The inherent difficulty of delivering such molecules across the intact BBB further compounds this challenge. Here, advanced drug delivery systems, particularly nanoparticles, hold significant promise. Strategies such as engineering nanoparticles with ligands that facilitate receptor‐mediated transcytosis across the BBB could ferry Mfsd2a agonists to their sites of action. However, the translation of these sophisticated systems requires careful optimization of their pharmacokinetics, safety profile, and large‐scale manufacturing processes. Furthermore, the exploration of plasma Mfsd2a as a biomarker, although highly promising, faces technical and translational hurdles. Current detection relies primarily on sophisticated techniques such as mass spectrometry or in‐house developed immunoassays, which are not yet standardized for routine clinical use. The development of a robust, high‐throughput, and validated assay—such as a clinically viable ELISA—is a prerequisite for large‐scale cohort studies and eventual clinical application. Key questions regarding its dynamic range, stability in plasma, and correlation with BBB integrity in humans remain to be fully addressed. The potential systemic effects of modulating Mfsd2a, given its expression in other barrier tissues, also warrant careful evaluation. A more nuanced discussion is required here. Mfsd2a is functionally critical in the placenta for transporting DHA to the developing fetus and in the retina for maintaining the blood–retinal barrier. Systemic pharmacological activation of Mfsd2a could therefore potentially interfere with these physiological functions, leading to unforeseen consequences in pregnancy or visual physiology. This risk necessitates a thorough investigation in toxicological studies and may drive the future development of brain‐specific targeting strategies to minimize systemic exposure and off‐target effects. We explicitly acknowledge that the current evidence is primarily mechanistic and prospective rather than demonstrative of confirmed therapeutic efficacy. Future work must focus on bridging this gap through large‐scale interventional studies and the exploration of Mfsd2a‐based biomarkers to stratify patients and guide therapy.

## 6 Conclusion

Mfsd2a, a key transport protein located on endothelial cells of the BBB, is indispensable for maintaining homeostasis in the brain. The significant downregulation of Mfsd2a expression after ischemic stroke is a key pathological link leading to BBB destruction, brain edema, intensified neuroinflammation and nerve injury. A large number of preclinical evidence strongly suggests that targeting Mfsd2a is a highly promising therapeutic strategy that can effectively stabilize the BBB, reduce brain injury, and improve the prognosis of neurological function.

Despite facing challenges in terms of in‐depth understanding of mechanisms, validation of large animal models, and clinical translation pathways, research in this field is advancing at an unprecedented pace. With the in‐depth exploration of the biology of Mfsd2a and its mechanism of action in stroke, as well as the continuous emergence of innovative treatment technologies and translational research methods, targeting Mfsd2a is expected to provide a novel, safe, and effective BBB‐centric neuroprotective therapy for patients with ischemic stroke in the near future, ultimately improving patients' quality of life and long‐term prognosis. Future research requires close interdisciplinary collaboration to jointly drive this promising target from the laboratory to clinical practice.

## Author Contributions

Jing Sun: Conceptualized the review. Jing Sun and Zhidong He: Literature search and data analysis. Zhidong He: First draft of the manuscript. Zhidong He under the supervision of Jing Sun: Figures were prepared. Jing Sun: Contributed to the study conception, carried out logic examination, and participated in the review of medical professional knowledge of the manuscript. Both authors contributed to the article and approved the final version.

## Funding

This study was supported by the Science and Technology Development Plan Project of Jilin Province in China (YDZJ202401696ZYTS).

## Ethics Statement

The authors have nothing to report.

## Consent

All the authors confirm that the work has not been submitted elsewhere for publication, either in whole or in part, and that its publication has been approved by all the coauthors.

## Conflicts of Interest

The authors declare no conflicts of interest.

## Data Availability

The data that support the findings of this study are available on request from the corresponding author. The data are not publicly available due to privacy or ethical restrictions.
